# Community-based Collaborative Care for Serious Mental Illness: A Rapid Qualitative Evidence Synthesis of Health Care Providers’ Experiences and Perspectives

**DOI:** 10.1007/s10597-025-01459-8

**Published:** 2025-03-27

**Authors:** Saira Abdulla, Sherianne Kramer, Lesley Robertson, Samantha Mhlanga, Campion Zharima, Jane Goudge

**Affiliations:** 1 Centre for Health Policy, School of Public Health, University of Witwatersrand, Private Bag X3 Wits 2050, Johannesburg, South Africa; 2 Department of Psychiatry, University of Witwatersrand, Johannesburg, South Africa; 3 Community Psychiatry, Sedibeng District Health Services, Sedibeng, South Africa

**Keywords:** Serious mental illness, Collaborative care, Case management, Multidisciplinary team, Review

## Abstract

Community-based collaborative care (CBCC) is an effective approach for addressing the needs of people with mental health conditions. However, even with the established components of CBCC in place, CBCCs effectiveness for serious mental illnesses (SMIs) remains unknown. This review aims to synthesize qualitative evidence of health care providers’ experiences of CBCC in order to identify key factors that facilitate or hinder collaboration in the specific context of SMIs. We searched databases to identify 3368 studies. The eligibility criteria included qualitative studies focusing on health care providers’ experiences in delivering a CBCC intervention for people with SMIs. Studies were included if they had at least 2 of 3 CBCC components: a multidisciplinary team, case management, and structured communication. Thematic analysis was used to synthesise the findings, and the Standards for Reporting Qualitative Research framework was used to assess the quality of included studies. The protocol is registered on Prospero. Of the 19 studies included in our review, 5 had achieved collaboration, which was driven by several key ingredients: the availability of on-site case managers and psychiatrists, or the psychiatrists’ willingness to travel to the site; the psychiatrists’ efforts in actively engaging and supporting the CBCC team; the primary care clinicians’ willingness to collaborate with the team and reduce traditional hierarchical engagement; the team’s understanding of CBCC; and case managers with strong interpersonal and professional skills. The inclusion of CBCC components do not guarantee collaboration. The findings emphasise the importance of on-site mental health specialists, clearly defined roles, and proactive providers in achieving collaboration.

**Supplementary Information:**

The online version contains supplementary material available at 10.1007/s10597-025-01459-8.

## Introduction

Community-based collaborative care (CBCC) aims to facilitate teamwork among mental health and primary health care (PHC) providers to improve the quality of services for health care users (HCUs) with mental and physical comorbidities (Ee et al., 2020). CBCC has been shown to reduce health care costs in the long-term (Miller et al., 2020), and is recommended by the World Health Organisation (WHO 2021).

While CBCC has been shown to be effective for people with common mental disorders (Archer et al., 2012; Gilbody, 2006; Kappelin et al., 2022; Neumeyer-Gromen et al., 2004), its effectiveness for serious mental illnesses (SMIs) is yet to be demonstrated (Byng et al., 2023; Reilly et al., 2013, 2024). For example, a recent quantitative review found only low-quality studies that showed improvements in health outcomes (Reilly et al., 2024). SMIs are defined by their enduring nature and associated functional impairment which causes substantial psychosocial disability, and comprise a range of disorders including personality, anxiety, eating, and depressive disorders as well as schizophrenia and bipolar disorder (Evans et al., 2016; National Institute of Mental Health, 2024). People with SMIs often experience inadequate social support, frequent hospitalisations, homelessness, unemployment, and incarceration (Evans et al., 2016). Importantly, considering the complexity of the conditions, CBCC does hold potential as a model of care for people with SMIs. While several theoretical models (Gunn et al., 2006; Reist et al., 2022; Wagner, 1998) have been used to operationalise CBCC, multidisciplinary teams, case management, and regular communication between providers are key components (Archer et al., 2012; Bower et al., 2006; Gilbody, 2006; Gilbody et al., 2003; Girard et al., 2019; Thota et al., 2012; Whitfield et al., 2023). These components are further described in Table 1.

**Table 1:** CBCC components

Components	Description
Multidisciplinary team	Intervention includes any three or more of the following categories of health care providers^a^: • Non-professional health care providers (lay health counsellors, community health workers)
Case management	Case manager or provider(s) who performs any of the following responsibilities: • Follow up of missed appointments • Monitoring HCU care • Coordinating input from various health care providers
Communication	Regular (weekly/monthly) case review meetings with at least 2 cadres of health care providers related to HCUs’ treatment and care and/or detailed referral/progress letters with follow-up communication

^a^While HCUs, carers, or care partners are considered part of the multidisciplinary team, they were not part of the eligibility criteria for this review as few studies included them as formal team members

(Archer et al., 2012; Bower et al., 2006; Gilbody, 2006; Gilbody et al., 2003; Girard et al., 2019; Thota et al., 2012; Whitfield et al., 2023)

Understanding barriers and facilitators as well as contextual factors is important if CBCC is to be implemented effectively. This review answers the research question: What are the key facilitators and barriers to CBCC for people with SMIs, based on health care providers’ experiences and perspectives? We aimed to synthesise global qualitative evidence of providers’ experiences of CBCC for SMIs, to identify barriers, facilitators, and contextual factors that influence whether collaboration takes place in a meaningful way. We discuss how facilitators could be adapted, and barriers overcome, especially in LMICs.

## Methods

We conducted a rapid systematic synthesis of qualitative evidence of CBCC for people with SMIs. The protocol is registered on Prospero [CRD42021250832], and is reported according to the Enhancing Transparency in Reporting the Synthesis of Qualitative Research (ENTREQ) guidelines (Tong et al., 2012).

### Eligibility Criteria

We included qualitative primary research studies of health care providers’ experiences of CBCC for community-dwelling adults^1^ 18 years or older with an SMI accessing PHC for treatment and care. Studies were included if the CBCC model included at least 2 of the three components in Table 1 and described the experiences of at least two different health care cadres. Studies about CBCC for people with medical comorbidities were included if management of the SMI was the primary focus. This review did not exclude articles based on language or geographical location.

### Search Strategy

PubMed, PsycINFO, Scopus, and reference lists (of included studies) were searched for eligible studies published since 2001. The year 2001 was selected based on the WHO’s, 2001 call for increased community-based mental health care, a key factor shaping the rise of CBCC in PHC settings. The search syntax (provided in Supplementary file 1) was based on three main constructs, namely SMIs, CBCC, and PHC, and was reviewed by a librarian. A Boolean search method using key operators (OR, AND) and truncation was used. Medical subject headings (MeSH) terms for key concepts were used to search PubMed and Scopus, and APA Thesaurus of Psychological Index Terms were used to search PsychINFO. Literature searches were conducted in March 2021 and updated in June 2023.

### Screening and Study Selection

References were exported into Covidence software and screened on title and abstract. To ensure consistent application of the inclusion criteria, all authors conducted independent, duplicate screenings in batches of 10 references, and differences in decisions were resolved through discussions. Consensus regarding the application of the criteria was reached after five rounds of screening and discussion. The remaining references were screened in duplicate by SA, CZ, and SM. Full text screening was conducted by SA.^2^ Where necessary, missing information were retrieved from study protocols and associated publications, or authors were contacted for further clarity.

### Data Extraction and Quality Assessment

Data were extracted from eligible studies by the lead author and reviewed by senior authors. Extracted information included study details (country, health care setting, and study design), SMI diagnoses and medical comorbidities, descriptions of care, experiences of care, and facilitators and barriers to collaboration. Data were initially extracted from three studies to pilot the extraction form. The Standards for Reporting Qualitative Research (SRQR) framework was used to assess the quality of included studies (O’Brien et al., 2014). Quality assessments were conducted by the lead author and each appraisal was reviewed by senior authors.

### Data Analysis and Synthesis

A thematic synthesis of the studies’ findings was conducted manually using an iterative process and inductive coding. Codes were generated according to the content and meaning of the findings for each study in succession. Emerging codes from subsequent studies were checked for applicability against previously coded studies. Codes were then examined for commonalities and organised to develop descriptive themes.

During analysis, the quality of communication between providers emerged as an important factor; communication that enabled multi-directional learning and shared responsibility for treatment and care, that contributed to a collective pool of expertise, and that reduced traditional power imbalances and hierarchies improved collaboration. We termed these types of interactions as ‘meaningful communication’.

We defined different levels of collaboration as follows:

- ‘Full collaboration’ is meaningful communication between three or more cadres. (If three or more cadres were engaging in this way, this suggests that the collaboration has become more systemic, i.e. part of the organisational culture.)
- ‘Partial collaboration’ is meaningful communication between two cadres only. (This may simply be enabled by personal relationships between individuals.)
- ‘Limited collaboration’ is a unidirectional flow of knowledge from one provider to another, with traditional hierarchical structures maintained.

## Findings

**Fig. 1:**
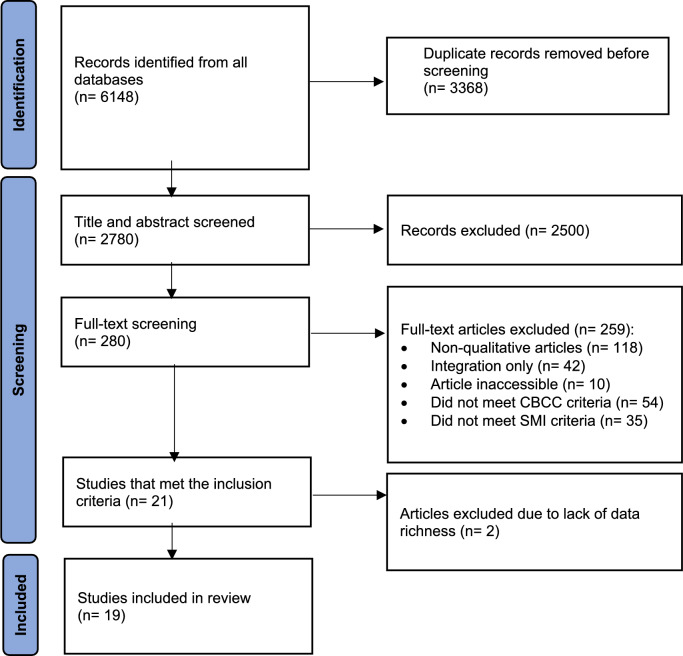
PRISMA diagram

The screening and selection process is summarised in the Preferred Reporting in Systematic Reviews and Meta-analyses (PRISMA) diagram (Fig. 1). After removing duplicates and screening titles and abstracts, 280 full texts were screened, and 21 studies met the criteria. Two articles (Miller et al., 2003; Ramanuj et al., 2018) were excluded during data extraction due to the paucity of their findings. Thus, 19 studies were included.

### Study Characteristics

Most studies (n = 17) were conducted in high-income countries (HICs), including the United States (US) (n = 10), the United Kingdom (UK) (n = 6), and Canada (n = 1). Two studies were conducted in middle-income countries (MICs) (India and China) (see Supplementary file 2). While no studies were conducted in low-income countries, several studies, including those from MICs, included low-income contexts where PHC sites were located in rural areas and/or primarily served low-income HCUs (n = 11). Studies focused on various SMIs, including major depression, anxiety, post-traumatic stress disorder, panic disorder, schizophrenia, and bipolar disorder. Sixteen studies scored 18 or more on the SRQR tool, which were considered high quality. The other three studies scored 16–17 and were rated as moderate quality.

### CBCC Components

The CBCC components and extent of collaboration in each study are presented in Supplementary file 3. All 19 studies had multidisciplinary teams and case management. In HICs, case managers were either PHC nurses, mental health nurses, social workers, mental health clinicians/psychologists, or medical assistants. In MICs, case managers were lay health counsellors (India) or community workers (China). There was regular communication between two or more providers in 15 studies, of which eight studies from diverse socio-economic and cultural contexts had meaningful communication. Five of these studies conducted in the US (n = 3), India (n = 1), and China (n = 1) achieved full collaboration (Al Achkar et al., 2020; Beck et al., 2018; Li et al., 2020; Ma & Saw, 2018; Pereira et al., 2011), while three studies in the US (n = 2) and UK (n = 1) achieved partial collaboration (Cerimele et al., 2014; Coupe et al., 2014; Nutting et al., 2008). Limited collaboration was found in 11 studies from the US (n = 7), UK (n = 5), and Canada (n = 1) where providers worked in silos (Baker et al., 2019; Batka et al., 2016; Beck et al., 2018; Bentham et al., 2011; Curran et al., 2012; Knowles et al., 2013, 2015; Lipschitz et al., 2017; Overend et al., 2015; Tanielian et al., 2016; Taylor et al., 2018; Wozniak et al., 2015).

### Barriers and Facilitators to Achieving Meaningful Collaboration

#### Physical Space

In several studies conducted in the US, there was insufficient physical space for additional staff (Bentham et al., 2011; Curran et al., 2012; Lipschitz et al., 2017): “*The people that we’re hiring aren’t going to be in the clinic because we don’t have space. We’re going to have to get creative in how to make sure they feel tied to those clinics even though they’re not physically located there*” [PHC clinician assistant] (Lipschitz et al., 2017). As a result, the placement of case managers varied across the US studies, including using vacant consultation rooms (Curran et al., 2012), providing telephonic care from a central location (Lipschitz et al., 2017; Nutting et al., 2008), and splitting their time between multiple sites (Bentham et al., 2011; Curran et al., 2012): “*I [case manager] work in three clinics. My car is my office*” (Curran et al., 2012).

There were several implications of case managers being off-site. Firstly, the provision of care was delayed: “*There was a lag time sometimes between when the referral was made and when I would get the referral. I think that affects a HCU’s willingness to participate*” (Curran et al., 2012). Secondly, there was insufficient time allocated to meet with HCUs (Bentham et al., 2011). Thirdly, the poor visibility of case managers hindered communication and rapport-building (Curran et al., 2012; Lipschitz et al., 2017): “*We were somewhat removed [in terms of location] from the [PHC] physicians, and I think it would have made communication a little bit easier, and just helped with having more presence*” [Case manager] (Curran et al., 2012). One study recommended the inclusion of case managers in face-to-face team meetings: “*I think what I would want to do is pair the care managers with the rest of the team; it’s very easy not to include them in the regular meetings because they’re not visible*” [Mental health chief] (Lipschitz et al., 2017).

#### Human Resources and Workload

Studies across various socio-economic settings in HICs and MICs experienced challenges with human resources (Al Achkar et al., 2020; Batka et al., 2016; Beck et al., 2018; Bentham et al., 2011; Curran et al., 2012; Knowles et al., 2013; Li et al., 2020; Lipschitz et al., 2017; Nutting et al., 2008; Overend et al., 2015; Pereira et al., 2011; Tanielian et al., 2016; Taylor et al., 2018). Limited staff resources impacted care: “*We don’t have enough staff to see people on a weekly basis. We have struggled to see suicidal patients weekly*” [Mental health specialist] (Tanielian et al., 2016). Some studies reported high staff turnover (Al Achkar et al., 2020; Batka et al., 2016; Beck et al., 2018; Wozniak et al., 2015), which made it difficult to provide continuity of care (Batka et al., 2016; Tanielian et al., 2016; Wozniak et al., 2015). “*There is a constant turnover in providers, so ensuring continuity of communication between providers is really difficult”* [PHC physician] (Batka et al., 2016).

In some studies conducted in the US, UK, and China, CBCC increased the workload of already overburdened PHC physicians (Bentham et al., 2011; Li et al., 2020; Lipschitz et al., 2017; Nutting et al., 2008; Tanielian et al., 2016), which impacted collaboration and communication (Knowles et al., 2013; Taylor et al., 2018): “*It’s taken days and days to get a response [from the PHC nurses], because they’re just too busy…*” [Case manager] (Knowles et al., 2013). PHC physicians were inundated with progress updates from case managers in studies from the US and UK (Nutting et al., 2008; Taylor et al., 2018): “*I get a telephone note that's sandwiched among the other 40 or 50 [computer alerts] with the abnormal stress test and the lung mass … I would like [to] get communication saying, ‘Hey listen, this person has a problem’*” [PHC physician] (Lipschitz et al., 2017). In a UK study, the workload made it difficult to dedicate enough time for consultations: “*In terms of appointment time, an 8- to 15-min allotment is not enough time*” [PHC physician] (Tanielian et al., 2016). Overburdened PHC physicians prioritised physical over mental health symptoms in another UK study (Overend et al., 2015). Case managers in studies conducted in the US and China were also overburdened and experienced burnout (Beck et al., 2018; Bentham et al., 2011; Li et al., 2020): “*We’ve parachuted some roles on top of people who already had other work to do. It felt burdensome, particularly for the social workers*” [Administrator] (Bentham et al., 2011).

Notwithstanding the above, increased workload burdens were not ubiquitous across studies (Curran et al., 2012; Knowles et al., 2013). In one study serving a population with significant mental health care needs in the United States (Curran et al., 2012), addressing HCUs’ needs improved health outcomes, leading to reduced clinic visits: “*My workload has not changed. Actually, it is my impression that patients actively enrolled in the treatment arm come in less*” [PHC physician] (Curran et al., 2012). In studies from the US and UK, case managers adopted a substantial portion of the workload (Curran et al., 2012): “*It frees us [PHC clinicians] up to concentrate perhaps more on the physical reviews, physical conditions*” [Nurse] (Knowles et al., 2013).

#### Registry Systems

Electronic health registry (EHR) systems were provided in five studies in the US and UK. EHR systems provide multiple longitudinal observations that enhance diagnostic accuracy and quality of treatment (Cerimele et al., 2014), and present the data in a way that encourages its real-time use for clinical decision-making (Bentham et al., 2011). Such systems were used by case managers to track and follow-up with HCUs (Cerimele et al., 2014; Ma & Saw, 2018). In one US study, the EHR provided access to information from anywhere at any time, which was “*especially important in this hard-to-reach population [i.e. low-income Asian immigrants in a metropolitan area] when no-shows are common*” (Ma & Saw, 2018). In another US study, while the EHR systems had utility, efficiency and continuity benefits, they were also limited by their functionality, thus requiring additional administrative work (Bentham et al., 2011). In the UK studies, case managers were not provided access to the EHR (Baker et al., 2019; Knowles et al., 2013), resulting in significant operational inefficiencies and communication barriers: “*I [case manager] feel quite blind by not having [access to] that system”* (Knowles et al., 2013)*.*

#### CBCC Knowledge

Effective collaboration among providers hinged on their understandings of the CBCC model and clarity about their roles across socio-economic contexts. Training was crucial (Bentham et al., 2011; Li et al., 2020; Wozniak et al., 2015): “*The training has helped me grow in my knowledge. And has helped me to get our program in a better shape. It’s helped the HCUs in the long run too*” [Administrator] (Bentham et al., 2011). In one Canadian study, case managers engaged in peer mentoring: *“I [senior case manager] sat in on [HCU] visits with [the other case managers]. Huge mentoring role. I don’t think it’s something that you can just jump in and say I’ll do this”* [Management] (Wozniak et al., 2015)*.* In the MIC studies, regular communication and support from the study intervention team improved provider engagement (Li et al., 2020; Pereira et al., 2011).

However, in some studies conducted in the US, UK, and Canada, CBCC training for PHC clinicians was either non-existent (Batka et al., 2016; Bentham et al., 2011; Coupe et al., 2014; Ma & Saw, 2018) or inadequate (Baker et al., 2019). Often PHC clinicians did not understand the CBCC framework (Baker et al., 2019; Coupe et al., 2014; Knowles et al., 2013; Taylor et al., 2018; Wozniak et al., 2015), the purpose of the case manager (Baker et al., 2019; Knowles et al., 2013), how to operationalise the case manager role in the clinic (Batka et al., 2016), or the role of different providers: “*[PHC physicians] in general are very confused about all of these mental health programs, and they don’t have any idea what’s what…they don’t know the difference between the depression care manager, me the embedded provider, and…the psychiatric nurse practitioner*” [Psychologist] (Lipschitz et al., 2017).

Similarly, some case managers in studies conducted in the US and UK lacked adequate knowledge of the CBCC framework and skills to effectively fulfil their roles (Baker et al., 2019; Batka et al., 2016): “*I don’t think I know what a case manager is*” [Case manager] (Knowles et al., 2013). Without prior experience in providing mental health care, some case managers (Batka et al., 2016; Knowles et al., 2013) and PHC physicians felt ill-equipped to treat HCUs with SMIs (Batka et al., 2016).

#### Building Cultural Competencies

Several studies conducted in the US and UK focused on diverse populations or ethnic groups from various socio-economic backgrounds (Al Achkar et al., 2020; Baker et al., 2019; Coupe et al., 2014; Curran et al., 2012; Ma & Saw, 2018). However, only two studies in the US reported on the cultural and language barriers: “*We have [PHC] clinicians… they never had any experiences working with our diverse population, specifically with [Asian and Pacific Islanders who had]* SMIs*. It’s something they don’t receive in school*” [Mental health specialist] (Ma & Saw, 2018). To address this, the two studies paired HCUs with case managers who shared a similar racial, cultural, or linguistic background, improving communication and understanding during appointments (Al Achkar et al., 2020; Ma & Saw, 2018). Integrating interpreters and peer workers into the care team strengthened the team's linguistic and cultural competencies (Al Achkar et al., 2020; Ma & Saw, 2018): “*[The] interpreter is a kind of support staff for us. He does interpreting for the Spanish-speaking patients and coordinating our schedules and calling our patients…”* [Case manager] (Al Achkar et al., 2020). However, this was time-consuming: *“The reason that the [visits] take so long is because… when you’re interpreting, you have that three-way conversation going on”* [Case manager] (Al Achkar et al., 2020)*.*

#### Case Managers’ Roles, Attributes, and Skills

Providers regarded case managers as important team members (Cerimele et al., 2014; Curran et al., 2012) who were “*vital to the success of the CBCC model*” (Bentham et al., 2011). In most studies, including MICs (n = 14), case managers coordinated care for HCUs. They also linked HCUs to community services in a UK study: *“…she [case manager] did everything she possibly could… She spoke to the people at Parkinsons UK to see if there was a network, an advice centre, and things I [HCU] didn’t know*” (Overend et al., 2015). In a US study, case managers played a key role in enabling communication between PHC physicians and psychiatrists: *“I have to call and remind the psychiatrist to fax over their notes to the PHC physician. One physician said: ‘We’ve been doing it for a year.’ Well they were sending it to the wrong clinic site”* [Case manager] (Nutting et al., 2008)*.* In the China study, case managers were from the communities they served, and were therefore better able to provide the team with context relating to HCUs, their families, and their living conditions (Li et al., 2020).

Various studies in the US, UK, and Canada highlighted essential skills and characteristics required for effective case management. Professional qualities included a background in CBCC, experience in medical (Wozniak et al., 2015) and/or mental health care provision (Beck et al., 2018; Wozniak et al., 2015), clinical management skills, and familiarity with community resources (Beck et al., 2018). Personal qualities included the ability to communicate effectively, and be well organized (Wozniak et al., 2015), confident (Wozniak et al., 2015), proactive (Baker et al., 2019; Curran et al., 2012; Lipschitz et al., 2017), and compassionate (Lipschitz et al., 2017). A US study found that case managers’ abilities to adapt to providers’ working styles, preferences, and schedules were important in facilitating communication: “*You have to learn the language of the medical team, you have to adjust to their pace. Your interaction may be two minutes long with that medical provider. What is it that you need to communicate to them and be respectful of them? The training has to focus on the culture of working in this type of setting”* [Case manager] (Al Achkar et al., 2020).

The significance of support to develop the necessary interpersonal and professional skills was emphasized in two studies (Batka et al., 2016; Pereira et al., 2011). The study from India found that using a non-specialist lay health counsellor with strong interpersonal and communication skills resulted in consistent communication with the PHC team (Pereira et al., 2011). While initially struggling to care for HCUs with complex presentations of SMIs, the case manager, supported by the psychiatrist, developed competence through ongoing learning and experience (Pereira et al., 2011). In another study conducted in the United States, a case manager was chosen for her interpersonal and telephonic skills despite lacking a clinical mental health background (Batka et al., 2016). Her struggle to build competence was attributed to insufficient professional development and support (Batka et al., 2016).

#### Role and Attitude of Psychiatrists

Studies from both HICs and MICs highlighted the essential role that psychiatrists played in supporting the CBCC team (Al Achkar et al., 2020; Cerimele et al., 2014; Coupe et al., 2014; Li et al., 2020; Ma & Saw, 2018; Pereira et al., 2011). Psychiatrists supported and helped upskill the case managers (Al Achkar et al., 2020; Cerimele et al., 2014; Coupe et al., 2014; Ma & Saw, 2018): “*Normally, a psychiatrist wouldn’t say, “What do you think of my suggestions pharmacologically?” But she’s [case manager] skilful enough, and we’ve worked long enough, she could say, “I think Zoloft is a good choice because I’m still not sure if the patient is breastfeeding” And I agree. It’s like a rich and synergistic experience where we’re thinking about patient care”* [Psychiatrist] (Al Achkar et al., 2020). Psychiatrists shared their expertise with PHC physicians, offering education and guidance in treating mental health (Al Achkar et al., 2020; Li et al., 2020; Ma & Saw, 2018; Pereira et al., 2011). This increased their levels of confidence and skills (Cerimele et al., 2014; Coupe et al., 2014; Nutting et al., 2008; Pereira et al., 2011).

There were several factors that enabled psychiatrists to successfully collaborate with PHC physicians and case managers in both MICs and HICs. In the MIC studies, psychiatrists who were willing to regularly visit clinics and were available telephonically often strengthened relationships with staff through respectful and timely interactions (Li et al., 2020; Pereira et al., 2011). In a US study conducted in a rural area, psychiatrists travelled considerable distances to improve collaboration: “*The problem is I’m working from hundreds of miles away from these folks. They sometimes can feel a bit detached from the fact that I’m actually a person on the other side of the system. We found it’s useful for us to drive, to go up there and meet with the PHC physicians”* [Psychiatrist] (Al Achkar et al., 2020). Studies from the US, India, and China found that the psychiatrists’ openness and willingness to assist staff resulted in PHC physicians voluntarily reaching out to psychiatrists for assistance (Cerimele et al., 2014; Li et al., 2020; Pereira et al., 2011): “*If I had any problem regarding patients, like if the dose had to be increased or patient is having minor side effects, I definitely consulted him [psychiatrist]…. He was always available on the telephone whenever I had any difficulties*” [PHC physician] (Pereira et al., 2011).

PHC physicians appreciated the psychiatrists’ feedback and treatment recommendations, thus learning from them (Al Achkar et al., 2020; Cerimele et al., 2014; Curran et al., 2012): “*We learn a ton from (the psychiatrist). He does a great job. He’s got kind of a little standard blurb that he puts in about each medication he’s discussed in the consult. He always gives us recommendations for things to explore with the patient in terms of their medical history or why they’re on certain meds or why they carry certain diagnoses. I’ve definitely learned a lot… from him*” [PHC physician] (Al Achkar et al., 2020). Psychiatrists also learned from case managers about the PHC context (Al Achkar et al., 2020; Cerimele et al., 2014): “*The big source of expertise is their knowledge of their local communities. Very often, we’re doing this consultation in distant communities. How that clinic system functions, the local environment, the culture, everything is very different. That’s some of the teaching that comes the other way*” [Psychiatrist] (Al Achkar et al., 2020). Psychiatrists who embraced team learning and acknowledged the expertise of the team facilitated meaningful collaboration rooted in mutual respect and shared knowledge (Al Achkar et al., 2020; Pereira et al., 2011). Thus each member of the multidisciplinary team contributed to the management of HCUs (Li et al., 2020).

Nevertheless, psychiatrists experienced some challenges in their roles. These included providing support in the context of shifting schedules (India) (Pereira et al., 2011), having to provide treatment recommendations with incomplete patient histories (US) (Cerimele et al., 2014), and not having enough time to provide support due to competing demands (Canada) (Wozniak et al., 2015). In a UK study, psychiatrists lacked understanding of the CBCC model and were unable to support case managers: “*I [case manager] was getting myself bogged down …and I’d go to [my] manager [psychiatrist] and I’d say … ‘I need help here’, and it was like ‘Read your manual’ and I felt like saying [shouts]: ‘You read the manual!*’” (Baker et al., 2019).

#### Buy-in and Endorsement of CBCC

Meaningful collaboration hinged on providers' readiness to work together as a team and share responsibility. One study in China found that the endorsement of the CBCC intervention by community and facility leadership led to buy-in and team engagement (Li et al., 2020). The study also found that PHC physicians had pre-existing relationships with the team, facilitating buy-in of the intervention (Li et al., 2020). Two studies from the US found that when providers fully embraced the CBCC philosophy, it had a transformative impact on the organizational culture (Bentham et al., 2011; Ma & Saw, 2018): “*[It] helps to lessen the hierarchical kind of structure that often occurs in a PHC clinic between mental health people and the PHC physicians*” [Social worker] (Bentham et al., 2011). Studies in the US found that PHC physicians who endorsed the intervention already believed in the importance of mental health (Curran et al., 2012), extended their consultation time to discuss mental health (Ma & Saw, 2018), identified HCUs who would benefit most from the intervention (Nutting et al., 2008), recognised the value of multidisciplinary provider teams (Beck et al., 2018; Bentham et al., 2011; Nutting et al., 2008), and worked with the case manager: “*…a good number of those [PHC physicians] bought into the intervention so I [case manager] had a steady flow of referrals. And they were pretty comfortable coming to get me to do an assessment [for the HCU]…”* (Curran et al., 2012).

However, this collaborative ethos was not embraced universally. A number of HIC studies in the US and UK found that PHC clinicians who believed that treating mental health was outside their scope of practice were resistant (Bentham et al., 2011; Curran et al., 2012; Knowles et al., 2013, 2015; Overend et al., 2015):*“It’s just you know, the old… war between primary care and mental health… they just don’t want to work together”* [Case manager] (Lipschitz et al., 2017). Some PHC physicians maintained the traditional hierarchical structure among staff in the US and Canada studies (Beck et al., 2018; Wozniak et al., 2015): “*It’s always been an “us and them” thing between nurses and doctors. Most of my doctors are around my age. When we first trained, we called them Sir and Doctor … they were always the boss… a lot of my doctors are still having trouble transitioning to the team idea”* [Case manager] (Wozniak et al., 2015). PHC physicians also struggled with relinquishing certain responsibilities and lacked trust in the CBCC intervention (Beck et al., 2018): *“I have difficulty of letting go, of saying, ‘I’m going to start you on this medication and I’m going to have somebody follow this.’ Part of my reluctance is I just don’t have that trust of the system yet”* [PHC physician] (Lipschitz et al., 2017)*.* Providers were also weary of losing their autonomy in a collaborative environment when they have traditionally practised independently (Nutting et al., 2008; Wozniak et al., 2015): “*I think [the physicians] find the principles of the study [sound], it’s just that they feel they’re giving up a little bit of autonomy*” [Management] (Wozniak et al., 2015).

As a result, studies from the US, UK, and Canada found that some PHC physicians restricted their involvement in CBCC by showing disinterest in receiving additional information on mental health, failing to refer HCUs to case managers (Knowles et al., 2013), ignoring or missing communication attempts from case managers (Knowles et al., 2013; Taylor et al., 2018; Wozniak et al., 2015), neglecting mental health (Overend et al., 2015), and disregarding treatment recommendations from psychiatrists (Wozniak et al., 2015). This presented a significant challenge to effective communication and teamwork among providers (Bentham et al., 2011; Knowles et al., 2013, 2015; Lipschitz et al., 2017; Taylor et al., 2018). Nonetheless, a study in the US found that case managers noted that a committed PHC nurse could act as a champion and facilitate referrals even when physicians were not enthusiastic: *“We had a couple of nurses that just really bought into it and referred and reminded the [PHC] physician, ‘This would be a really good patient (for CBCC)’”* [Case manager] (Curran et al., 2012)*.*

## Discussion

In this review, we synthesized qualitative evidence on health care providers’ experiences and perspectives of CBCC for SMIs in order to identify key facilitators and barriers to collaboration. Out of the 19 studies included in this review, eight achieved meaningful communication (i.e. full collaboration in five studies (including MIC studies) and partial collaboration in three studies). Meaningful communication was driven by a combination of several key ingredients: the availability of on-site case managers and psychiatrists, or the psychiatrists’ willingness to travel to the site; the psychiatrists’ efforts in actively engaging and supporting the CBCC team; the PHC clinicians’ willingness to collaborate with the team and move away from traditional hierarchical engagement; the team’s understanding of CBCC along with clarity on roles and responsibilities; and case managers with strong interpersonal and professional skills (see Supplementary file 4).

These results are similar to findings from a qualitative systematic review by Overbeck and colleagues (Overbeck et al., 2016) on CBCC for common mental disorders (i.e. depression and anxiety) in HICs. Overbeck and colleagues found that buy-in, on-site location of case managers to support communication, and the social and professional skills of the case manager were key to facilitating CBCC (Overbeck et al., 2016). They also found that reimbursements for extra work, feedback on the effectiveness of the programme, and PHC physicians who advocated for collaboration were facilitators to CBCC implementation (Overbeck et al., 2016). Our review details the efforts made by providers in achieving collaboration and sheds light on the complexities and dynamics of collaboration among health care providers, offering a more nuanced understanding of the factors that enable or hinder effective CBCC for SMIs.

While Overbeck and colleagues (Overbeck et al., 2016) included studies with psychiatrists as part of the CBCC team, they did not explore their collaborative role in-depth. Our review, however, found that psychiatrists are essential to supporting the CBCC team, perhaps due to the complex nature of SMIs (Demyttenaere et al., 2004; Iseselo et al., 2016; Swartz et al., 1998). In LMICs, the shortage of mental health specialists is a significant barrier to delivering mental health care services (Rathod et al., 2017). According to the World Health Organisation’s Global Health Observatory Data Repository, the availability of psychiatrists in LMICs is estimated to range from 0.01 to 1.52 per 100,000 people (World Health Organization, 2019), with the majority located in urban areas (Rathod et al., 2017). CBCC models in LMICs therefore include remote psychiatrists who provide support to case managers and PHC providers in providing mental health services (Whitfield et al., 2023). In comparison, our review emphasised the importance of accessible psychiatrists, either on-site or those committed to regular site visits despite distance, to support PHC staff and facilitate meaningful collaboration. However, majority of the studies included in our review were conducted prior to the rise of the hybrid work model that emerged after the COVID-19 pandemic. This raises the question as to whether meaningful collaboration can occur without in-person contact.

A quantitative review by Bower and colleagues (2006) found that case managers with a professional background in mental health improved depression symptoms for HCUs. Similarly, our review found that case managers who had a background in mental health, as well as strong interpersonal skills excelled in their roles. However, our review also found that while prior experience in mental health is important, case managers who initially struggled in their roles developed competencies over time through experience, on-the-job learning, and support from psychiatrists. Similar to Overbeck and colleagues’ (2016) review, we found that scepticism among PHC clinicians decreased when case managers demonstrated relational and professional competence.

Similar to other quantitative reviews, we found that case management is provided by various cadres. These include lay health counsellors, community health workers, nurses, mental health providers, allied health care providers, or distributed between different health care providers (Bower et al., 2006; Gilbody, 2006; Whitfield et al., 2023). The studies in India and China suggests the acceptability of non-professional health care providers, who shared the same cultural and linguistic background as the HCU population, in performing case management functions for HCUs with SMIs (Li et al., 2020; Pereira et al., 2011; Shinde et al., 2013). Similarly, Overbeck and colleagues’ (2016) study highlighted the importance of ‘*skilled and culturally sensitive providers*’ as key facilitators of CBCC. Upskilling lay health counsellors is not a new phenomenon; numerous studies in LMICs have adopted this approach in CBCC interventions to alleviate the shortage of health care specialists and to mitigate service delivery bottlenecks, especially for mild to moderate mental health disorders (Buttorff et al., 2012; Petersen et al., 2012; Shahmalak et al., 2019). Services provision by lay health workers can improve health outcomes and cost-savings (Kangovi et al., 2020; Landers & Levinson, 2016), highlighting the effectiveness and practicality of upskilling non-specialist providers for case manager roles in LMICs (Keynejad et al., 2022).

We also found that PHC clinicians who lacked interest or training in CBCC or mental health often prioritized HCUs’ physical health over mental health concerns, even when the latter were more severe. Similarly, Overbeck and colleagues’ (2016) findings revealed that competing priorities and limited participation hindered meaningful collaboration. Our review found that while most studies focused on collaboration between case managers and PHC physicians, nurses emerged as champions of the CBCC programme, stepping in effectively when physicians showed little interest or engagement. Empowering nurses with greater decision-making authority, including the ability to prescribe psychotropic medication, a practice adopted in some LMICs (Joshi et al., 2014; SANAC, 2023), can expand the reach of mental health care services.

## Recommendations

This study highlights the factors that facilitate or hinder meaningful collaboration, primarily in HICs. In Table 2, we provide recommendations for adapting CBCC in LMICs, taking into account resource constraints, local contexts, and tele-psychiatry. The findings of this review are important for policy makers and researchers, providing opportunities to inform policies and guide further research in addressing the complexities of implementing CBCC for SMIs across diverse settings.

**Table 2:** Recommendations for adapting CBCC in LMICs

Strategy	Meaningful collaboration in HICs	Adaptation to LMICs
Capacity building	• Training of various cadres: o Understanding CBCC o Skills development for treating and managing SMIs • On-the job training provided by psychiatrists	• Strengthen local capacity with training for *all* health care providers (including non-specialist providers) before and during CBCC implementation in order to: o Develop mental health care competencies o Understand CBCC interventions o Sustain and improve CBCC delivery
Psychiatrists	• On-site psychiatrists (or psychiatrists willing to travel to sites regularly despite distance) are key to facilitating collaboration • Support team by providing: o Education, o Explanatory notes for treatment recommendations, and o Availability telephonically	Mitigate specialist shortages through: • *Hybrid service delivery:* combine tele-psychiatry with on-site consultations where possible. (This would require technological solutions to suit local infrastructures, such as low-bandwidth tele-health for areas with limited internet access) • *Hybrid supervision*: telephonic and in-person site visits to facilitate collaboration with team where possible • *Distributed care*: Enable mental health clinicians to serve multiple locations, perhaps running a service once a week in a PHC clinic, overseen by a supervising remote psychiatrist
PHC clinicians	• Provide mental health care • Are champions of the intervention • Communicate with team	• Empower nurses with decision-making authority, prescription capabilities, and responsibilities often held by psychiatrists in HICs • Include nurses and other non-physician providers supervised by remote or visiting mental health specialists to deliver mental health care and collaborate with allied and mental health providers (psychiatrists, psychiatric nurses, psychologists, case managers)
Case managers	• Case managers include PHC nurses, mental health nurses, social workers, mental health clinicians/psychologists, or medical assistants • Case management roles sometimes divided across multiple providers	• Incorporate lay health counsellors into the CBCC teams, supervised by psychiatrists/psychologists, mental health practitioners to ensure quality of care
Local context	• Providers with the same culture/ language as HCUs • Use of interpreters	• Train providers to engage with diverse populations in LMICs to ensure cultural awareness • Integrate traditional and indigenous healers into the health care team, and respect local cultural practices and beliefs • Respect cultural expressions and understandings of SMIs
HER	• EHR facilitates information sharing and exchange • Supportive tool for case management functions	• Pen and paper method in resource constraint settings

## Limitations

This review has several limitations. 1) This review employed a rapid review approach, relying on three electronic databases for the literature search. This approach may have led to the exclusion of some relevant studies, however, data saturation was reached, suggesting a comprehensive coverage of the topic. 2) While the review focused on qualitative studies to explore the experiences and perspectives of providers on CBCC, there is a need for further high-quality quantitative studies to examine the effectiveness of CBCC for SMI, especially in LMICs. 3) The review considers low-income contexts within studies from HICs and MICs, however, the inclusion of only two studies from MICs limits the generalizability of our findings across different cultural contexts and settings. 4) This review only focused on providers’ experiences of CBCC, however, future primary research should expand on HCUs perspectives, as well as the socio-economic and cultural contexts in order to provide a more comprehensive synthesis of the factors that hinder or facilitate CBCC for SMIs. 5) Studies that included urban, rural, and suburban sites or diverse populations did not draw comparisons between these groups. Furthermore, various studies lacked contextual information, limiting comparisons across socio-economic or cultural contexts. 6) This review found that on-site providers were key to facilitating collaboration. However, the rapid increase in hybrid and online models of care and communication post the COVID pandemic requires further exploration of CBCC using tele-modalities.

## Conclusion

This review identified the key ingredients that facilitate or hinder meaningful communication and collaboration for SMIs in PHC settings. The inclusion of CBCC components does not guarantee meaningful communication. The findings emphasise the importance of on-site mental health specialists, clearly defined roles, and proactive providers. While global research on the effectiveness of CBCC is expanding, there remains a significant need for further research to bridge the gaps in our understanding, particularly in adapting CBCC approaches for SMIs in the context of LMICs.

## Supplementary Information

Below is the link to the electronic supplementary material.

Supplementary file1 (DOCX 15 KB)

Supplementary file2 (DOCX 19 KB)

Supplementary file3 (DOCX 31 KB)

Supplementary file4 (DOCX 69 KB)

## Data Availability

The search strategy is available in Supplementary file 1. Study characteristics, CBCC components, and role-related facilitators and barriers to achieving collaboration are available in Supplementary files 2, 3 and 4.

## Abbreviations

CBCC: Community-based collaborative care
EHR: Electronic health registry
ENTREQ: Enhancing transparency in reporting the synthesis of qualitative research
HCU: Health care user
LMIC: Low- and middle-income country
MIC: Middle-income country
PHC: Primary health care
SMIs: Serious mental illnesses
SRQR: Standards for reporting qualitative research
WHO: World health organisation

## Footnotes

1 People with SMIs living within the vicinity of PHC services.

2 Weekly meetings with the research team were conducted at every step of the review process, including screening, data extraction, quality assessment, and data synthesis to ensure rigour, consistency, and accuracy during the review process.
