# Supplementary material for: Community-based Collaborative Care for Serious Mental Illness: A Rapid Qualitative Evidence Synthesis of Health Care Providers’ Experiences and Perspectives
Source: Community Ment Health J. 2025 Mar 27;61(6):1195–207. doi: 10.1007/s10597-025-01459-8 (PMC12228660; doi:10.1007/s10597-025-01459-8)
Supplement: Supplementary file 1 — Supplementary file1 (DOCX 15 KB) [file 10597_2025_1459_MOESM1_ESM.docx]

## Additional file 1. Search Strategy

( "Serious mental illness" OR "Severe mental illness" OR "Serious mental disorders" OR "Severe mental disorders" OR "Psychiatric disorders" OR "Schizophrenia Spectrum and Other Psychotic Disorders" OR "Personality Disorders" OR "Anxiety Disorders" OR "Bipolar and Related Disorders" OR "depression" OR "Depressive Disorder" OR "Mood Disorders" OR "Severe depression" OR "Eating Disorders" OR "Anorexia Nervosa" OR "Bulimia Nervosa" ) AND ( "collaborative care" OR "collaborat*" OR "interprofessional collaboration" OR "cooperative behavior" OR "intersectoral collaboration" OR "integration" OR "interprofessional relations" OR "collaborative mental health care" OR "coordinated care" OR "integrated care teams" OR "care manager" OR "case managers" OR "case management" OR "task sharing" OR "team work" OR "team approach" OR "communication" OR "multidisciplinary teams" OR "multidisciplinary care" OR "multidisciplinary" OR "patient care team*" OR "delivery of health care, integrated" ) AND ( "PHC" OR "primary health care" OR "primary healthcare" OR "primary medical care" OR "community-based health care" OR "community-based healthcare" OR "community-based care" OR "integrated PHC" OR "community-care setting" )

**Note:** The search strategy was initially designed to include all study designs. However, during data extraction, the review team decided to include qualitative study designs only. As such, we did not filter qualitative study designs in the advanced search strategy.
