# Supplementary material for: Community-based Collaborative Care for Serious Mental Illness: A Rapid Qualitative Evidence Synthesis of Health Care Providers’ Experiences and Perspectives
Source: Community Ment Health J. 2025 Mar 27;61(6):1195–207. doi: 10.1007/s10597-025-01459-8 (PMC12228660; doi:10.1007/s10597-025-01459-8)
Supplement: Supplementary file 2 — Supplementary file2 (DOCX 19 KB) [file 10597_2025_1459_MOESM2_ESM.docx]

**Additional file 2. Description of Studies**

| **Author(s), year** | **Country & Setting** | **Aim** | **Condition** | **Data collection methods** | **Participants** | ***SRQR score** |
| --- | --- | --- | --- | --- | --- | --- |
| Achkar, 2020 | United States   - Rural - Three PHC sites serving Spanish-speaking HCUs | To investigate the feasibility of systematic case reviews for depression. | Depression | Interviews | - PHC physicians - Ancillary staff - Psychiatrists - Case managers | 20 |
| Baker 2019 | United Kingdom, England   - Urban and rural - Three PHC sites in varied regions with diverse ethnicities | To assess intervention theory and to identify the barriers and facilitators to delivering the model as intended. | - Schizophrenia - Bipolar | - Interviews - Consultations recordings | - PHC clinicians - Psychiatrists - Case managers - HCUs - Family carers | 19 |
| Batka 2016 | United States   - PHC clinics at six large military installations | To examine experiences of integrating mental health treatment within PHC clinics. | - Depression - Post-traumatic stress disorder | Interviews | - PHC clinicians - Social workers - Psychologists - Psychiatrists - Case managers - HCUs | 20 |
| Beck 2018 | United States   - Rural, suburban and urban - PHC clinics across eight states | To describe the implementation of a large-scale CBCC intervention | - Depression - Diabetes - Cardiovascular disease | - Interviews - Observational data | - Physician consultant - Psychiatrist - Case manager | 19 |
| Bentham 2011 | United States   - Post Katrina disaster recovery setting - Low income, uninsured, and predominantly African-American HCUs. | To describe experiences of a collaborative care mental health approach for treating depression and anxiety. | - Depression - Anxiety - Post-traumatic stress disorder | Interviews | - PHC clinicians - Administrators - Community health worker - Social workers - Psychiatrist - Case managers | 17 |
| Cerimele 2014 | United States   - Safety net PHC clinics serving low-income HCUs | To understand views and current practices on diagnosis and management of HCUs with bipolar disorder in PHC settings using a CBCC model. | - Bipolar disorder - Substance use disorder | Focus groups | - Psychiatrists - Psychiatric nurses | 18 |
| Coupe 2014 | United Kingdom   - PHC sites serving various populations of mixed socio-economic status in different areas. | To explore the impact of CBCC on professional relationships, its feasibility in PHC and implementation barriers and facilitators. | - Depression - Anxiety | Interviews | - PHC physician - Supervisors (research team mental health clinicians) - Case managers | 21 |
| Curran 2012 | United States   - Seventeen clinics in four areas diverse in size, HCU population, and insurance mix | To explore the facilitators and barriers to implementing and sustaining CBCC. | - Major depression - Anxiety disorders - Panic disorder - Posttraumatic stress disorder - Alcohol use | Interviews | - PHC clinicians - Administrator - Case managers | 20 |
| Knowles 2013 | United Kingdom, England   - Large PHC Trust | To assess the implementation of CBCC principles in routine care for depression and other chronic conditions and identify barriers and facilitators of adopting CBCC. | - Depression - Diabetes - Coronary heart disease - Chronic obstructive pulmonary disease | Interviews | - PHC nurses - Case managers | 17 |
| Knowles 2015 | United Kingdom, England   - 15 PHC practices | To explore HCUs’ and providers’ perspectives of CBCC. | - Depression - Diabetes - Coronary heart disease - Chronic obstructive pulmonary disease | Interviews | - PHC clinicians - Case managers - HCUs | 20 |
| Li, 2020 | China   - Rural - Villages clinics that provide PHC serving elders | To examine how CBCC was implemented and identify facilitators and barriers for its more widespread implementation. | - Depression - Hypertension | Focus groups | - Village doctors - Case managers - Psychiatrists | 18 |
| Lipschitz 2017 | United States   - Two Veterans Affairs medical centres | To evaluate the benefits and barriers of having a dedicated case manager in comparison to an embedded-only model of CBCC | - Depression - Anxiety | Interviews | - Management - PHC clinicians - Mental health providers - Case manager | 21 |
| Ma 2018 | United States   - Metropolitan area - Community mental health clinic serving low-income Asian immigrants | To better understand the facilitators and barriers of integrated care in a multilingual behavioural health setting. | - SMI unspecified | - Focus groups - Interviews | - Management - PHC clinicians - Mental health providers - HCUs | 19 |
| Nutting 2008 | United States   - Small, mixed-payer PHC practices | To examine the experience of providers in integrating care management into enhanced care of depressed HCUs. | - Major depressive disorder - Dysthymia. | Interviews | - PHC physicians - Mental health specialists - Case managers | 20 |
| Overend 2015 | United Kingdom, England   - Urban and rural - Three sites serving older HCUs (65+) | To explore HCUs’ and professionals’ views to gain an understanding of depression management. | - Major Depression - Diabetes - Heart disease | Interviews | - PHC physicians - Case managers - HCUs | 20 |
| Pereira 2011 | India   - Urban and rural - Twelve private and twelve public PHCs in low-resourced settings | To describe the experiences of integrating the CBCC model into PHC. | - Moderate-severe common mental disorders | Interviews | - PHC physicians - Ancillary staff - Clinical specialists - Case managers | 17 |
| Tanielian 2016 | United States   - PHC clinics at six large military installations | To examine barriers to mental health care faced by military members in accessing services. | - Depression - Post-traumatic stress disorder | Interviews | - PHC clinicians - Psychologists - Social workers - Psychiatrists - HCUs | 20 |
| Taylor 2018 | United Kingdom, England   - Urban and rural - Three sites serving older HCUs (65+) | To explore views on CBCC and how this model could be implemented at scale. | - Major depression - Diabetes - Heart disease | Interviews | - PHC physicians - Case managers - HCUs | 20 |
| Wozniak 2015 | Canada   - Four nonmetropolitan PHC network sites | To evaluate the implementation of an efficacious collaborative care model in community-based PHC networks | - Major Depression - Diabetes | Interviews | - Management - Research team - Psychiatrists - Endocrinologist - Internists - Case managers | 18 |
