# Supplementary material for: Community-based Collaborative Care for Serious Mental Illness: A Rapid Qualitative Evidence Synthesis of Health Care Providers’ Experiences and Perspectives
Source: Community Ment Health J. 2025 Mar 27;61(6):1195–207. doi: 10.1007/s10597-025-01459-8 (PMC12228660; doi:10.1007/s10597-025-01459-8)
Supplement: Supplementary file 3 — Supplementary file3 (DOCX 31 KB) [file 10597_2025_1459_MOESM3_ESM.docx]

**Additional file 3. CBCC components, barriers and facilitators, and process outcome**

| **Author Year**  **Country** | **CBCC components** | **Barriers** | **Facilitators** | **Extent of collaboration** |
| --- | --- | --- | --- | --- |
| Achkar, 2020  United States | Multidisciplinary team   - PHC physicians - Ancillary staff (medical assistants, interpreters) - Psychiatrist - Case manager   Case management   - Case manager had limited mental health experience - Located on-site   Communication   - Regular case review meetings between case manager and psychiatrist - Communication between case manager and PHC physicians - Communication between psychiatrist and PHC physicians | PHC physicians  Discomfort and unpreparedness in managing SMI cases  Psychiatrists   - Located off-site and thus felt isolated from team - Training of new team members due to turnover   Case managers and PHC physicians  Initially reported limited knowledge of mental disorders and team-based care  Interpreter  3-way conversations (between case manager, HCU and interpreter) were time consuming | PHC physicians   - Often newly qualified and in their first job, thus open to CBCC - Learnt from listening to case manager and interacting with, and reading notes from psychiatrists - Over time, began referring more HCUs to case managers   Psychiatrists   - Approached team with humility - Travelled long distance to build relationships with PHC clinicians - Coached case managers on presenting recommendations to PHC clinicians   Case Manager   - Matched with HCUs based on language - Adapted to faster pace of PHC - Learned how to build relationships and adapted to PHC clinicians’ diverse work and communication styles - Coordinated care   Case managers and PHC physicians  Developed competence through experience and on-the-job learning  Interpreter  Availability of interpreters | Full collaboration |
| Baker, 2019  England | Multidisciplinary team   - PHC physicians - Psychiatrist - Case managers     Case management   - Case managers mental health experiences varied - Limited physical health experience - Located on-site   Communication   - Little communication between case manager and psychiatrist - Little communication between case manager and PHC clinicians - No communication between psychiatrist and PHC physicians | PHC physicians   - Did not understand the case manager role - Disinterest in CBCC for mental health   Psychiatrist   - Competing demands hindered support to case managers - Assumed the case manager was competent - Inadequate training   Case managers   - Inadequate training - Unfamiliar with PHC systems - Marginalised from PHC teams - Poor recording in shared records - Some lacked confidence to engage with PHC physicians - Limited care coordination   Multidisciplinary team  Lacked understanding of CBCC model | Case managers   - Some sought support from research team - Some were proactive in approaching PHC physicians   Psychiatrist   - Some understood the CBCC model and participated in the intervention | Limited collaboration |
| *Batka, 2016;  Tanielian, 2016  United States | Multidisciplinary team   - PHC clinicians - Psychiatrists - Psychologists - Case managers - Social workers   Case management   - Case managers had no prior experience in mental health - Location varied (specific site/central location)   Communication   - Communication between case manager and psychiatrist unknown - Little communication between case manager and PHC clinicians | PHC clinicians   - Shortage of clinicians - Provider turnover made it challenging for case managers to build trust and ensure continuity of care   Psychiatrists   - Most doubted PHC physicians’ readiness to address mental health. - Some believed that HCUs require care offered in specialty settings   Primary and mental health specialists   - Concerns about developing and maintaining relationships with each other due to lack in trust - Uncertain about the role and purpose of case managers   Case manager   - Struggled to manage HCUs with SMIs - Limited care coordination | PHC clinicians  Most PHC clinicians felt competent to address mental health in their clinical settings due to prior experience in treating mental health. | Limited collaboration |
| Beck, 2018  United States | Multidisciplinary team   - PHC physicians - Internist/family clinician specialist - Psychiatrist - Case managers - Social workers   Case management   - Case managers’ mental and physical health care experiences varied   Communication   - Regular case review meetings between case manager, psychiatrist, and specialist consultant - Communication between case managers and some PHC physicians | Sites with low engagement between staff:  PHC physicians   - Provider turnover in some sites - Traditional conceptualisation of care - Perceived lack of benefit of intervention - Resisted CBCC (in sites with no previous exposure to case managers) - Reluctant to cede some control of HCU care to case manager - Poor morale and buy-in   >>Some sites struggled with low engagement throughout the CBCC intervention.  Case managers   - Resisted documenting HCU data in both the case management tracking system and EHRs - Burnout from managing mental and medical comorbidities, and arranging social services - Miscalculation of funding for case management | Sites with high engagement between staff:  PHC physicians   - Positioning of intervention as a PHC physician-led model vs case manager-led model - Buy-in for those who already believed in the importance of mental health - Referred HCUs to CBCC intervention   >>Some sites started with low engagement, but over time gained understanding and trust in the intervention    Case managers   - Establish rapport quickly with HCUs - Exercise independent judgement - Awareness of local services - Coordinated care   Social workers   - Some sites used social workers to facilitate HCUs’ access to community resources   Multidisciplinary team   - Clinic culture supports team-based models, shared records, and shared responsibility for HCU care - Team dynamics of cohesiveness, trust, and valuing each other’s contributions - Increased level of interaction between providers | Highly engaged sites: full collaboration  Low engagement sites: Limited collaboration |
| Bentham, 2011,  United States | Multidisciplinary team   - PHC clinicians - Psychiatrist - Case managers - Social workers - Community health worker   Case management   - Case managers had mental health experience - Located on-site   Communication   - Case manager supported by on-site psychiatrist who provides treatment recommendations. (This interaction was not explored in the article) - Little communication between case managers and PHC clinicians | PHC clinician   - Did not have enough training - Did not consistently use protocols and screening tools - Referral challenges due to constantly changing referral process - Screening competed with other clinical objectives during consults and was time consuming - Resistance due to perceptions that mental health care was not within scope of their practice - Did not update the team about HCUs’ progress   Case managers   - In some sites, case management functions were distributed among various clinicians who already had existing responsibilities - Burdensome EHR system - Limited space and time to meet HCUs - Post-disaster setting affected case management as HCUs did not have stable housing/telephones | PHC clinician   - Valued having access to the case manager who provided information between HCU visits - Appreciated the support of accessible psychiatrists - Comfortable in:   -screening for mental health,  -prescribing antidepressants,  -referring to specialty mental health services  Case manager   - Some sites had a dedicated case manager - Coordinated care   Social workers   - Appreciated having their expertise valued and discussing HCU care with other clinicians - Reported reduced hierarchical structure between mental health and PHC physician   Multidisciplinary providers  Viewed CBCC model as a tool to prevent patients from being missed. | Limited collaboration |
| Cerimele, 2014  United states | Multidisciplinary team   - PHC physicians - Psychiatrists - Case managers   Case management   - Case managers had mental health care experience - Located on-site   Communication   - Regular case review meetings between case manager and remote psychiatrist - Joint meetings with HCU, PHC clinicians and case manager for complex cases - Communication between case manager and PHC physicians - Over time, communication between psychiatrist and PHC physicians increased? | Psychiatrist   - Sometimes had to make treatment recommendations in the absence of complete history of HCU | Over time, PHC physicians   - Developed trusting relationships with pro-active case manager - Perceived positive HCU outcomes when referred to case manager for CBCC - Receptive to psychiatrists’ treatment recommendations - Became comfortable and competent in treating mental health - Engaged with psychiatrists about treatment   >>Initiated several steps of treatment before referring HCUs to case manager for CBCC  Psychiatrists   - Adapted their specialty mental health care skills to PHC - Provided education to case managers and PHC clinicians - Addressed PHC clinicians’ questions about mental health and explained reasoning behind treatment recommendations - Relied on case managers to learn about HCUs and develop treatment recommendations   Case managers   - Highly trained and pro-active - Engaged with HCUs’ social network - Repeated follow-up of at-risk HCUs who defaulted care, and successfully re-engaged HCUs in CBCC care - Learnt from psychiatrists on how to care for HCUs - Improved competency over time - Coordinated care | Partial collaboration |
| Coupe, 2014  United Kingdom | Multidisciplinary team   - PHC physicians - Psychiatrists - Psychologists - Case manager   Case management   - Case managers had mental health experience - Based off-site   Communication   - Regular supervision meetings between case manager and psychiatrist - Limited communication between case manager and PHC physicians (using progress reports) | PHC physicians   - Most lacked understanding of the CBCC framework - Were mostly unaware of case managers' involvement and work with their HCUs - Reported little impact on their routine work or professional relationships - Most had limited/no communication with case managers. - Did not reciprocate case managers’ efforts to communicate - Direct contact with case manager was rare, typically during HCU crises   Psychiatrist  Perceived GPs’ lack of time  Case managers   - Not provided access to HCU records in some sites - Different IT systems at various sites - Based off-site made it difficult to build relationships with PHC physicians | PHC physicians   - Few understood CBCC because of interest or prior experience - More open to suggestions from case managers when informed these are based on meetings with psychiatrist - Positive feedback from HCUs led to belief in some value of the intervention   Psychiatrist   - Provided support, ongoing learning, and confidence boost for case managers - Satisfied with the case managers’ skills for delivering treatment, even for complex HCUs   Case managers   - Some had pre-existing relationships with PHC clinicians - Attempted to identify PHC clinicians’ preferred method of communication at the beginning of the trial - Coordinated care   Psychiatrist and case managers   - Received training and understood CBCC - Good professional relationships with each other - Impressed with each other’s skills - Monitor HCU progress jointly | Partial collaboration |
| Curran, 2012  United States | Multidisciplinary team   - PHC clinicians - Psychiatrist - Psychologist - Case manager   Case management   - Case managers’ mental health care experiences varied - Location varied   Communication   - Regular case review meetings between case manager and remote psychiatrists - Communication between case managers and PHC physicians (in hallways, lunchtime, or during staff meetings) | PHC clinicians   - Some uninterested in mental health and did not buy-in to CBCC - Discomfort treating mental health - Poor engagement with part-time PHC clinicians - Communication with case managers unsatisfactory, some wanted more, others felt communication was too much - PHC clinicians did not feel like CBCC was worth putting effort into as grant funding was limited   Case managers   - Limited space for case managers in many sites and part time case managers hindered communication and referrals - Difficulty paying for case managers once grant funding ends | PHC clinicians   - Fifty percent self-identified as champions of the intervention and already believed in mental health as a priority - Nurses emerged as programme champions, despite being initially excluded from the intervention - Little to no increase in workload - Positive HCU outcomes increased enthusiasm and referral activity - Referrals worked best when case managers were on-site - Pre-existing presence of a mental health clinician and CBCC services for another disorder facilitated the use of the case manager   Case managers   - Proactive, warm, engaging, and visible - Increased ‘face time’ with physicians bolstered implementation - Coordinated care | Limited collaboration |
| *Knowles, 2013, 2015  England | Multidisciplinary team   - PHC clinicians - Psychiatrist - Case manager   Case management   - Case managers had mental health care experience - Limited physical health care experience - Located on site   Communication   - Two joint meetings between case manager, PHC nurse, and HCU | PHC clinicians   - No CBCC buy-in - Reluctance towards joint meetings - Did not find information provided by case managers on mental health necessary - Did not see the necessity of conversations with case managers and did not want feedback - GPs had little involvement   Case managers   - Struggled to engage with PHC clinicians - Not equipped to deal with comorbidities, thus struggled with their role - No access to EHR - Limited care coordination   Case managers and PHC clinicians   - Did not understand CBCC or their roles in the intervention - Maintained division between mental and physical health care | Case managers   - Gained confidence in managing SMIs with complex comorbidities - Care coordination and sharing information with PHC clinicians   Case managers and PHC clinicians   - Over time, improved communication resulting in better understanding of HCUs’ conditions | Limited collaboration |
| Li, 2020  China | Multidisciplinary team   - Village doctor - Psychiatrist (consultant) - Case managers   Case management   - In-person - Conduct home visits for assessments of HCUs’ social stressors and support - Educate HCU and family - Support HCUs’ treatment adherence   Communication   - Weekly in-person meetings between village doctor and case manager.   The psychiatrist joined monthly by telephone.   - Village doctors call the psychiatrists for additional consultations. | Village doctor   - Worked overtime - Low reimbursement   Case manager   - Reduced resistance from other HCUs - Role overload - Intervention added extra burden thus tiring - Low reimbursement   Psychiatrist   - Travelling to village was time consuming | Village doctor   - Had pre-existing relationship with team   Case manager   - Received continuous support from project staff - Had support of village leadership, enabling buy-in from HCUs   Psychiatrist   - Responded immediately to communication from village doctor   Village doctor and case manager   - Worked in the village for a long time and had a good understanding of the village - Developed trusting relationships with HCUs and found that the intervention increases positive HCU outcomes - Created treatment plan together   Village doctor and psychiatrist   - Psychiatrists conduct diagnostic assessment, and in consultation with the village doctor initiate antidepressant treatment   All providers   - Geographic proximity - Shared understanding and appreciation of intervention and team approach - Felt that each member had unique strengths, improving HCUs’ management (i.e. case managers know more about the patients and their families’ living conditions and village doctor and psychiatrist had their own specialist knowledge and skills) - Felt that leadership (village and hospital) cared about the study (i.e. integration and CBCC) - Wanted to support their leaders by being part of the team - Attended training sessions (improving mental health knowledge for village doctors and case managers)   >>Team coordination, collaboration, shared care, joint planning and problem-solving, frequent communication | Full collaboration |
| Lipschitz, 2017  United States | Multidisciplinary team   - PHC clinicians - Psychiatrist - Psychologist - Case managers   Case management  Case managers often located off-site  Communication   - On-site psychiatrist supervises case managers (This interaction was not explored in the article) - Communication between case manager and PHC physicians | PHC physicians   - Did not know the difference between case managers and mental health clinicians - Unclear roles and responsibilities - High workload and turnover - Conflicts with case manager regarding division of labour and work tasks - Struggled to let go of responsibility for all aspects of HCU care - Difficulty trusting case managers - Inundated with progress reports   Sites with case managers   - Challenge finding case managers with interpersonal qualities - No space for case managers on-site, thus invisible and unintegrated with PHC teams, in turn affecting collaboration   Sites without case managers   - Case management functions less organized - Monitoring and referral activities depend on the preferences and capabilities of individual PHC clinicians | PHC clinicians  Reached out to onsite mental health clinicians for same day consultations with their at-risk HCUs  Case manager  Care coordination on sites with case manager | Limited collaboration |
| Ma, 2018  United States | Multidisciplinary team   - PHC clinicians - Psychiatrist - Case manager   Case management   - Located on-site - Experience in mental health   Communication   - Formal and informal communication channels - Joint meetings between PHC physicians, HCU and case managers (This interaction was not explored in the article) - Regular case conferences with multidisciplinary team - EHR to share information, coordinate care, and monitor progress | Multidisciplinary team   - All clinicians had different understanding of programme and how it should work - Challenges in creating a shared vision of integrated care, clarifying roles and responsibilities between behavioural health and primary care, and developing a mutual understanding of the administrative procedures - Increased workload in integrated care setting - Co-location: - No space for co-location of services - Different protocols in two organisations   Mental health and PHC clinicians   - Concerns about the current reimbursement system that does not compensate for increased workload in integrated care setting   PHC clinicians   - Lack of training - Difficulty recruiting multilingual clinicians | PHC clinicians   - Initiated frequent communication with case managers - Acknowledged importance of behavioural health interventions - Dedicated more time to understanding the HCUs’ mental health conditions - Viewed their patients as shared within the team   Mental health clinicians   - Took time to educate case managers and PHC physicians   Case managers   - Bilingual/bicultural case managers aid language translation - Willingness to work with PHC physicians - Conducted more follow-ups with HCUs on PHC clinicians’ recommendations   Multidisciplinary team   - Continuous access, exposure and education on each other’s work - Exchanged information and improved communication - Discuss HCUs treatment goals and progress - Daily check-ins to ensure HCUs are not overlooked within the system - Coordinated care - Ensure services from different parties were provided to meet the HCUs’ needs   >>Improvements in organizational culture | Full collaboration |
| Nutting, 2008  United States | Multidisciplinary team   - PHC clinicians - Mental health specialists - Case managers   Case management   - Case managers’ experiences varied - Located in a central location serving multiple practices   Communication   - Regular case review meetings between case manager and mental health specialist - Communication between case manager and PHC physicians | PHC clinicians   - Increased workload due to   - time needed to add another member to team  - referring and communicating with the case manager   - Lack of reimbursement - Few were sceptical about whether case managers were worth the cost - Resistant providers concerned about infringement of their autonomy | PHC clinicians   - High acceptance of case management - Appreciated treatment recommendations - Most valued regular feedback about HCUs from case managers - After working with case manager and seeing their patients improve, sceptical clinicians became more enthusiastic - Believed that working with case manager promoted teamwork, expanded information available to physician, and improved care   >> enhancing physician-patient relationships   - Post-trial, clinicians became more selective of who they referred for case management, i.e., more complex HCUs   Mental health specialist   - Enthusiastic about providing oversight for the case managers   Case managers   - Established early face-to-face relationship with clinicians - Some asked clinicians how they would like to communicate - Assurance and support from specialist boosted case managers’ confidence in dealing with HCUs - Coordinated care   Post-trial, sites adjusted case manager locations based on needs   - Some sites had case managers on-site - Others adopted hybrid arrangements - Some case managers split their time among various practices | Partial coordination |
| *Overend  2015; Taylor 2018  England | Multidisciplinary team   - PHC clinicians - Mental health specialist - Case manager   Case management   - Case managers had mental health care experience - Located off-site   Communication   - Limited communication between case manager and PHC physicians (reports/ telephonic calls) - Case manager supervised by a mental health specialist | PHC physicians   - Lack understanding of CBCC - Reluctance to identify mental health conditions due to limited time and treatment options, thus prioritizing physical symptoms - Limited time for collaboration with case manager due to workload.   Case managers  Perceived physicians as too busy based on their work hours and volume of reports, this limiting opportunities for collaboration | Case manager   - Most understood the intervention, and their role - Received training - Linked HCUs to other social organisations - Coordinated care   PHC clinicians and case managers   - Recognised importance to liaise with each other - Offered different perspectives on HCUs’ health care needs in turn reducing ‘blind spots’ in care   Psychiatrist  Received training | Limited collaboration |
| Pereira, 2011  India | Multidisciplinary team   - PHC physician - Ancillary staff - Psychiatrist - Case manager   Case management   - Case managers had no previous mental health care experience - Belonged to the community - Located on-site - Regular peer supervision   Communication   - Psychiatrist provides supervision to case manager and support to team - Communication between case manager and PHC doctor | PHC Physicians   - Several doctors only sought consultation from psychiatrist if facilitated by case manager - Older doctors’ reluctance to participate due to preconceived notions of depression   Psychiatrists  Provided uneven support due to doctors changing shifts or unavailable due to other commitments  Case managers  Initially struggled with their role and dealing with complex HCUs  Health assistants  Caseload challenges | PHC doctors   - Acceptance of programme evolved over time as their rapport with, and trust of the CMs competence grew - Observed positive clinical outcomes of HCU - Endorsed importance of psychiatrist in the programme - Over time, referred unscreened HCUs to health assistants - Discussed treatment of HCUs with CM   Psychiatrists   - Support case managers in dealing with difficult cases - Overcame PHC clinicians’ reluctance to participate: - regular presentation of the program performance - reviewing the program protocol regularly - regular visits to the clinics, - availability on the phone - building strategic relationships through timely, respectful, and supportive interactions with team - uplifted doctors to support the programme and prescribe medication, thus increasing their confidence   Case managers   - Coordinated care - Conducted home visits/sent letters to HCUs - Good listener, empathetic, supportive - Over time, became competent in their role   Health assistants   - Conducts screening - Additional assistants allocated in busy clinics - Polite and friendly - Helped clinicians sharpen their diagnostic abilities and treatment - Went beyond role and offered advice, explored HCUs’ perspectives about their conditions, and aided in relieving distress | Full collaboration |
| Wozniak, 2015  Canada | Multidisciplinary team   - PHC clinicians - Psychiatrists - Endocrinologist - Internists - Case managers   Case management   - Case managers had limited experience in mental health care - Some located off-site - Two nurses split the role in one site   Communication   - Regular case review meetings between case manager and specialists - Communication between case manager and PHC physicians | PHC clinicians   - Varied participation - Existing culture limited participation:   - autonomy of clinicians  - traditional hierarchical relations of physician-nurse (case manager)   - Limited comfort in practising CBCC - ‘Unsolicited treatment recommendations’ from study specialists (relayed by case managers)   Psychiatrist  Supervisors’ degree of satisfaction depended on quality of relationship with case manager  Diabetes specialists/endocrinologists   - Lack of time - Lack of administrative support - Medico-legal issues and compensation issues - Providing care from a distance   Case managers   - Challenging finding case managers with the right mix of professional and personal qualities - Turnover in two sites - Discontinuity of case managers during the intervention - Limited care coordination | Psychiatrists   - Pre-existing relationships between specialists and the physicians - Would participate in the study again   Case managers   - Built relationships with HCU - Enjoyed work - Rewarding to see HCUs improve - Learned by shadowing or being mentored by other case managers | Limited collaboration |

*In cases where multiple papers by the same author or on the same intervention were published, these studies were combined to provide a more comprehensive picture of the findings.
