# Supplementary material for: Community-based Collaborative Care for Serious Mental Illness: A Rapid Qualitative Evidence Synthesis of Health Care Providers’ Experiences and Perspectives
Source: Community Ment Health J. 2025 Mar 27;61(6):1195–207. doi: 10.1007/s10597-025-01459-8 (PMC12228660; doi:10.1007/s10597-025-01459-8)
Supplement: Supplementary file 4 — Supplementary file4 (DOCX 69 KB) [file 10597_2025_1459_MOESM4_ESM.docx]

**Additional file 4. Role-related facilitators and barriers to achieving collaboration**

| **Facilitators in studies with full collaboration** | **Barriers in studies with limited collaboration** |
| --- | --- |
| **PHC providers** |  |
| - Endorsed CBCC (Al Achkar et al., 2020; Beck et al., 2018; Ma & Saw, 2018; Pereira et al., 2011) - Competence grew with experience (Al Achkar et al., 2020) - Initiated frequent communication with case managers (Ma & Saw, 2018) - Prior exposure to CBCC models (Beck et al., 2018) and team (Li et al., 2020) - Viewed HCUs as shared within the team (Ma & Saw, 2018) - Leadership buy-in enabled their support of the intervention (Li et al., 2020) - Attended training, improving mental health knowledge (Li et al., 2020) - Dedicated more time to understanding their HCUs’ mental health (Ma & Saw, 2018) | - High turnover (Beck et al., 2018; Lipschitz et al., 2017; Tanielian et al., 2016; Wozniak et al., 2015) - Increased/high workload (Lipschitz et al., 2017; Nutting et al., 2008; Taylor et al., 2018) - Existing hierarchical structures (Wozniak et al., 2015) - Traditional outlook of care (Beck et al., 2018) - No training (Bentham et al., 2011) - Unclear roles and responsibilities (Baker et al., 2019; Batka et al., 2016; Lipschitz et al., 2017) - Poor understanding of CBCC (Baker et al., 2019; Coupe et al., 2014; Knowles et al., 2013; Taylor et al., 2018) - Difficulty trusting case manager (Lipschitz et al., 2017) - Reluctance to cede some control of HCU care to case manager/autonomy (Beck et al., 2018; Lipschitz et al., 2017; Nutting et al., 2008; Wozniak et al., 2015) - No buy-in (Beck et al., 2018; Curran et al., 2012; Knowles et al., 2013, 2015; Wozniak et al., 2015) - No interest in mental health and/or CBCC (Baker et al., 2019; Bentham et al., 2011; Curran et al., 2012; Knowles et al., 2013) - Did not reciprocate case managers’ engagement (Coupe et al., 2014; Knowles et al., 2013, 2015) - Prioritized physical symptoms over mental health (Overend et al., 2015) - Inundated with progress reports (Lipschitz et al., 2017) - Communication with case managers either too little or too much (Curran et al., 2012). - ‘Unsolicited treatment recommendations’ from study specialists (Wozniak et al., 2015) - Grant funding limitations hindered participation (Curran et al., 2012) - Sceptical that case managers were worth the cost (Nutting et al., 2008) |
| **Case Managers** |  |
| - On-site (Al Achkar et al., 2020; Ma & Saw, 2018; Pereira et al., 2011). - Linguistically/culturally compatible case managers (Al Achkar et al., 2020; Ma & Saw, 2018) - Members of the community (Li et al., 2020; Pereira et al., 2011) - Leadership buy-in enabled their support of the intervention (Li et al., 2020) - Attended training, improving mental health knowledge (Li et al., 2020) - Competence grew with experience (Al Achkar et al., 2020; Pereira et al., 2011) - Coordinated care (Al Achkar et al., 2020; Beck et al., 2018; Ma & Saw, 2018; Pereira et al., 2011) - Interpersonal and professional skills:   - Good listener, empathetic, supportive(Pereira et al., 2011)   - Experience with population, aware of local services (Beck et al., 2018)   - Adapted to the pace of PHC (Al Achkar et al., 2020)   - Adapted to PHC physicians’ work and communication styles (Al Achkar et al., 2020) | - High turnover (Wozniak et al., 2015) - Challenge finding case managers with interpersonal qualities (Lipschitz et al., 2017; Wozniak et al., 2015); passive; lacked confidence (Baker et al., 2019); burnout (Beck et al., 2018) - Inadequate training (Baker et al., 2019) - Located off-site (Coupe et al., 2014) - Limited physical space (Bentham et al., 2011; Curran et al., 2012; Lipschitz et al., 2017) - Burdensome EHR system (Batka et al., 2016; Beck et al., 2018; Bentham et al., 2011; Coupe et al., 2014) - No access to HCU records (Coupe et al., 2014; Knowles et al., 2013) - Poor recording in shared records (Baker et al., 2019) - Time consuming responsibilities (Beck et al., 2018) - Lacked knowledge of PHC systems (Baker et al., 2019) - Poor understanding of CBCC (Baker et al., 2019; Knowles et al., 2013) - Excluded from PHC teams (Baker et al., 2019) - Struggled with role/lacked competence (Knowles et al., 2013; Tanielian et al., 2016) - Case management functions burdensome (Bentham et al., 2011) - Discontinuity of case managers during the intervention (Wozniak et al., 2015) - Limited care coordination (Baker et al., 2019; Knowles et al., 2013; Tanielian et al., 2016; Wozniak et al., 2015) |
| **Psychiatrists** |  |
| - Attended training (Li et al., 2020) - Leadership buy-in enabled their support of the intervention (Li et al., 2020) - Approached team with humility (Al Achkar et al., 2020; Pereira et al., 2011) - Travelled long distance to meet PHC clinicians on-site (Al Achkar et al., 2020) - Regular on-site visits (Ma & Saw, 2018; Pereira et al., 2011) and telephonic availability (Li et al., 2020; Pereira et al., 2011) - Educated and supported team (Al Achkar et al., 2020; Ma & Saw, 2018; Pereira et al., 2011) - Uplifted doctors to support the programme and prescribe medication, thus increasing their confidence (Pereira et al., 2011) | - Inadequate training and poor understanding of CBCC (Baker et al., 2019) - Uncertain about the role and purpose of case managers (Batka et al., 2016) - Lack of time (Wozniak et al., 2015) - Located off-site (Wozniak et al., 2015) - Competing demands thus unable to support case managers (Baker et al., 2019) - Assumed competency of case manager (Baker et al., 2019) - Doubted physicians’ readiness to address mental health; lack of trust (Batka et al., 2016). |
